# Surgical treatment of spontaneous brainstem hemorrhage: A case report

**DOI:** 10.1097/MD.0000000000018430

**Published:** 2019-12-20

**Authors:** Guangshan Hao, Zhentao Xu, Jianxin Zhu

**Affiliations:** Department of Neurosurgery, Liaocheng People's Hospital, Liaocheng, China.

**Keywords:** massive, microneurosurgery, spontaneous brainstem hemorrhage

## Abstract

**Rationale:**

The improvement of microneurosurgery and neuroimaging, as well as neuronavigation and neurophysiological monitoring, enables neurosurgeons to safely and accurately resect lesions on the brainstem.

**Patient concerns:**

A 54-year-old man, with 2-year history of hypertension, presented with sudden loss of consciousness for 1.5 hours.

**Diagnoses:**

Spontaneous brainstem hemorrhage.

**Interventions:**

We performed posterior fossa decompression together with hematoma evacuation in the super early stage for the patient.

**Outcomes:**

The patient regained normal spontaneous breathing function after surgery. And he needed help for daily activities with hemiplegia of right limb at three-month follow-up.

**Lessons:**

The hematoma evacuation together with posterior fossa decompression in the super early stage maybe a good treatment for patients in a deep coma with a large hematoma at the dorsal side.

## 1 Introduction

Hemorrhage on the brainstem, which is regarded as the vital center of the body, has always been a difficult clinical problem due to its high mortality. The most common of these is pontine hemorrhage, with an incidence of 6% to 7% and a mortality up to 40% to 50%.^[1,2]^ With the development of microneurosurgery, doctors have tried surgical treatments for brainstem hemorrhage. Brainstem hemorrhages secondary to cavernous malformations are much more benign and patients can get a good prognosis through microneurosurgery treatment.^[3]^ Instead, the surgical treatment of spontaneous brainstem hemorrhage is still controversial with pro and con.^[4–7]^ The ongoing continuous developments of microsurgical techniques, intraoperative neurophysiological monitoring, and neuronavigation make surgical treatment of brainstem hemorrhage easier and safer. Moreover, with the development of neurological intensive care, neural stem cells and rehabilitation, survivors will expect a better prognosis. However, there are currently few reports on the surgical treatment of spontaneous brainstem hemorrhage and no standardized guidelines. Here we report one case of patient with severe spontaneous brainstem hemorrhage who underwent surgical treatment.

## 2 Case presentation

**Figure 1:**
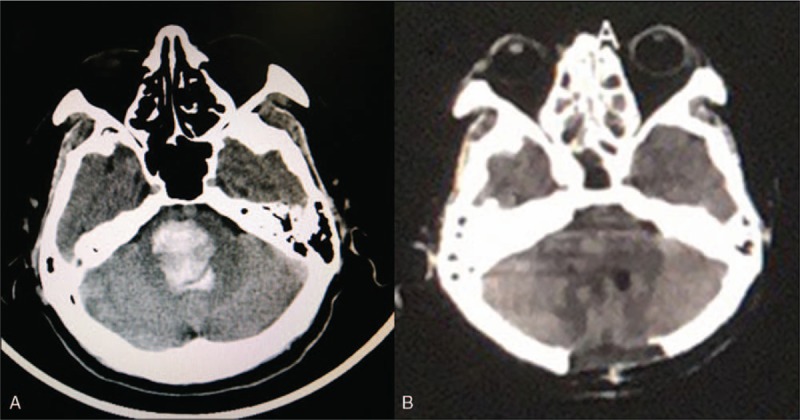
Preoperative CT scan showed a large hematoma mainly located on pontine extending to fourth ventricle (A); Postoperative CT showed mostly removal of the hematoma (B).

A 54-year-old man, with 2-year history of hypertension, presented with sudden loss of consciousness for 1.5 hours. On his arrival to hospital, he was in a deep coma (Glasgow Coma Scale (GCS) of 3) with unstable vital signs (Respiratory Rate: 3bpm, Blood Pressure: 190/104 mmHg). Bilateral pupil was equal (diameter: 2 mm) and non-reflective to light. Mechanical ventilation was given immediately after admission. The head Computed Tomography (CT) scan showed a large hematoma (about 11 ml) mainly located on the dorsal side of pons extending to fourth ventricle and slightly enlarged lateral ventricles (Fig. 1A). With the family's consent, we performed hematoma evacuation together with posterior fossa decompression through a suboccipital midline approach for the patient 3 hours after the onset. A small pathological artery was coagulated gently with low bipolar cautery and no cerebrovascular malformations were observed during the surgery. On the third day after surgery, the patient regained the stable spontaneous breathing function and CT was performed showing that the hematoma was mostly removed (Fig. 1B). The patient was transferred to the rehabilitation ward 2 weeks later. And he needed help for daily activities with hemiplegia of right limb at three-month follow-up (Glasgow Outcome Scale: 3).

## 3 Discussion

Spontaneous brainstem hemorrhage is a rapid, devastating stroke, with a high mortality up to 40% to 50%.^[1,2]^ At the onset, rapid accumulation of hematoma on brainstem leads to disruption of normal anatomy causing irreversible damage. However, the mass effect of the hematoma and the secondary damage of the blood cell degradation products can be alleviated by surgery. Moreover, hematoma on brainstem degrades very slowly due to its lack of glial cells. Fortunately, some case reports have provided evidence of the effectiveness of microneurosurgery treatment for patients with primary brainstem hemorrhage, for it not only reduces mortality but also improves functional outcome.^[4,6,8]^ However, the data about the surgery treatment enrolled in previous studies is really little and there is no special guideline applied to it from American Heart Association/American Stroke.

To provide optimal treatment, neurologists should first evaluate patients quickly and accurately. In recent studies, it has been reported that the prognosis is highly dependent on the severity of clinical manifestations and some imaging indicators.^[8]^ The classification of brainstem hemorrhage based on CT performance proposed by Chung and Park in 1992 has been widely accepted, in which the hemorrhages were roughly divided into 4 types: small unilateral tegmental, basal tegmental, bilateral tegmental and massive.^[9]^ Survival rate is the highest in small unilateral tegmental type and lowest in massive type. According to the study of predictors mentioned above and some case reports of microneurosurgery treatment together with our experiences, the current recommended surgical indications mainly based on the degree of coma and imaging result are as follows^[4,6,8]^: 1) GCS ≤ 8; 2) volume≥ 5 ml (concentrated and superficial). However, when the hematoma volume is over 10 ml, patients may get a poor outcome no matter treated conservatively or surgically.^[4]^ Time is also a very important factor. Evacuation of the hematoma as soon as possible can not only relieve the compression effect but also avoid a series of secondary damage. Some other reports also suggested a better prognosis of patients underwent surgery within 6 hours after the onset.^[6]^

In this case, the massive hematoma caused respiratory failure. If the treatment was conservative treatment or palliative extraventricular drainage, then the patient may eventually have died due to prolonged compression on the brainstem. However, the patient had a short onset, and the regular hematoma was located on the dorsal side extending to fourth ventricle. The normal tissues of the brainstem were not severely damaged, however, the compression of the hematoma was the major risk factor. Fortunately, the patient regained normal spontaneous breathing function after surgery, and the patient's left limbs could perform some certain voluntary activities at 3-month follow-up. We hope that the hematoma evacuation together with posterior fossa decompression in the super early stage maybe a good treatment for patients in a deep coma with a large hematoma at the dorsal side. The small sample size is a limit of this study, and more further research should be done to develop a reasonable surgical guideline for treatment of spontaneous brainstem hemorrhage.

## Author contributions

**Resources:** Guangshan Hao, Zhentao Xu, Jianxin Zhu.

**Writing – original draft:** Guangshan Hao.

**Writing – review & editing:** Zhentao Xu, Jianxin Zhu.

## References

[1] Murata Y Yamaguchi S Kajikawa H Relationship between the clinical manifestations, computed tomographic findings and the outcome in 80 patients with primary pontine hemorrhage. J Neurol Sci 1999;167:107–11. 10521548 10.1016/s0022-510x(99)00150-1

[2] Wijdicks EFM StLouis E Clinical profiles predictive of outcome in pontine hemorrhage. Neurology 1997;49:1342–6. 9371919 10.1212/wnl.49.5.1342

[3] Sandalcioglu IE Wiedemayer H Secer S Surgical removal of brain stem cavernous malformations: surgical indications, technical considerations, and results. J Neurol Neurosurg Psychiatry 2002;72:351–5. 11861694 10.1136/jnnp.72.3.351 PMC1737795

[4] Zhang HT Chen LH Bai MC Anterior subtemporal approach for severe upper pontine hematomas: a report of 28 surgically treated cases. J Clin Neurosci 2018;54:20–4. 29779725 10.1016/j.jocn.2018.04.063

[5] Kim JY Bae HJ Spontaneous intracerebral hemorrhage: management. J Stroke 2017;19:28–39. 28178413 10.5853/jos.2016.01935 PMC5307946

[6] Shrestha BK Ma L Lan ZG Surgical management of spontaneous hypertensive brainstem hemorrhage. Interdiscip Neurosur 2015;2:145–8.

[7] Hemphill JC Greenberg SM Anderson CS Guidelines for the management of spontaneous intracerebral hemorrhage a guideline for healthcare professionals from the American Heart Association/American Stroke Association. Stroke 2015;46:2032–60. 26022637 10.1161/STR.0000000000000069

[8] Tao CY Li H Wang JJ Predictors of surgical results in patients with primary pontine hemorrhage. Turk Neurosurg 2016;26:77–83. 26768872 10.5137/1019-5149.JTN.12634-14.1

[9] Chung CS Park CH Primary pontine hemorrhage - a New Ct-Classification. Neurology 1992;42:830–4. 1565238 10.1212/wnl.42.4.830

